# Evaluation of a Virtual Reality-Based Open Educational Resource Software

**DOI:** 10.1177/23821205241242220

**Published:** 2024-04-02

**Authors:** Silvia Würstle, Lisa-Marie Spanke, Niklas Mehlhase, Gail Stanley, Jonathan Koff, Stavros Dimitriadis, Sarah König, Alexander Hann

**Affiliations:** 1 Department of Internal Medicine II, Infectious Diseases, University Hospital Frankfurt, Goethe University Frankfurt, Frankfurt, Germany; 2 Department of Internal Medicine II, University Hospital rechts der Isar, School of Medicine, Technical University of Munich, Munich, Germany; 3 Interventional and Experimental Endoscopy (InExEn), Department of Internal Medicine II, Gastroenterology, University Hospital Würzburg, Würzburg, Germany; 4 Institute of Medical Teaching and Medical Education Research, University Hospital Würzburg, Würzburg, Germany; 5 Department of Internal Medicine, 12228 Yale School of Medicine, New Haven, CT 06520, USA; 6 Department of Gastroenterology, University Hospital Coventry and Warwickshire, Coventry, CV2 2DX, UK

**Keywords:** virtual reality, medical education, open educational resource software, educational tools

## Abstract

**OBJECTIVES:**

Virtual reality (VR) teaching methods have potential to support medical students acquire increasing amounts of knowledge. EVENT (Easy VR EducatioN Tool) is an open educational resource software for immersive VR environments, which is designed for use without programming skills. In this work, EVENT was used in a medical student VR course on pancreatic cancer.

**METHODS:**

Medical students were invited to participate in the course. Before and after VR simulation, participants completed a multiple-choice knowledge assessment, with a maximum score of 10, and a VR experience questionnaire. The primary endpoint compared pre- and post-VR simulation test scores. Secondary endpoints included usability and factors that could affect learning growth and test results.

**RESULTS:**

Data from 117 of the 135 participating students was available for analysis. Student test scores improved by an average of 3.4 points (95% CI 3.1-3.7, *P *< 0.001) after VR course. The secondary endpoints of gender, age, prior knowledge regarding the medical subject, professional training completed in the medical field, video game play, three-dimensional imagination skills, or cyber-sickness had no major impact on test scores or final ranking (top or bottom 25%). The 27 students whose post-VR simulation test scores ranked in the top 25% had no prior experience with VR. The average System Usability Scale score was 86.1, which corresponds to an excellent outcome for user-friendliness. Questionnaire responses post-VR simulation show students (81.2% [95/117]) interest in more VR options in medical school.

**CONCLUSIONS:**

We present a freely available software that allows for the development of VR teaching lessons without programming skills.

## Introduction

Medical students are challenged by the need to acquire an increasingly vast amount of knowledge. Simulation-based education, and particularly immersive virtual reality (VR) simulations, have potential as promising modalities to revolutionize medical education.^1–4^ VR environments are computer-generated 3D environments in which students can immerse themselves and interact with the environment via a head-mounted display (HMD). The introduction of commercially available, low-cost HMDs led to the widespread application of VR techniques in various areas of medical treatment and education, such as anatomy and neuroanatomy education,^5–7^ laboratory skills,^8^ endoscopy,^9,10^ echocardiography,^11^ medical obstetrical education,^12^ communication strategies,^4,13^ optimizing the institutional climates of medical schools and health systems,^14^ and addressing the social determinants of health.^15^ However, until now, preparing VR teaching courses has required time-intensive resources and programming skills. In contrast, EVENT (Easy VR EducatioN Tool)^16^ is a VR educational software designed to require limited time and no programming skills. Educational courses based on this open-source software have not yet been studied for effectiveness or usability.

VR-based education has been shown to contribute to student learning by increasing their motivation through a varied learning format, and by using dynamic visualization to better imagine or understand medical concepts and associations.^7,9,17–19^ Learning itself, as well as long-term retention of learning, might be facilitated by linking theory to various stimuli and physical movements,^2,3,17,18,20^ and students might improve their self-assessment through embedded quizzes. On the other hand, challenges, such as technical problems, little prior experience with VR or video games, poor spatial awareness, motivation, excitement from the immersive experience itself, or cyber-sickness (motion sickness by using the VR device) could impair the learning experience.^2^

Therefore, we conducted a medical teaching course using the new open-source EVENT software, and we investigated: (1) its effectiveness on test scores and learning growth, (2) its usability, and (3) factors that might influence successful VR-based learning.

The topic of pancreatic cancer is not covered in detail in medical school. Therefore, this area of knowledge was optimal to evaluate the increase in knowledge through a VR teaching unit. Specifically, the knowledge imparted included the etiology, epidemiology, classification, symptoms, diagnosis, histopathology, differential diagnosis, therapeutic approaches, and prognosis of pancreatic cancer.

## Materials and methods

### Software

**Figure 1.**
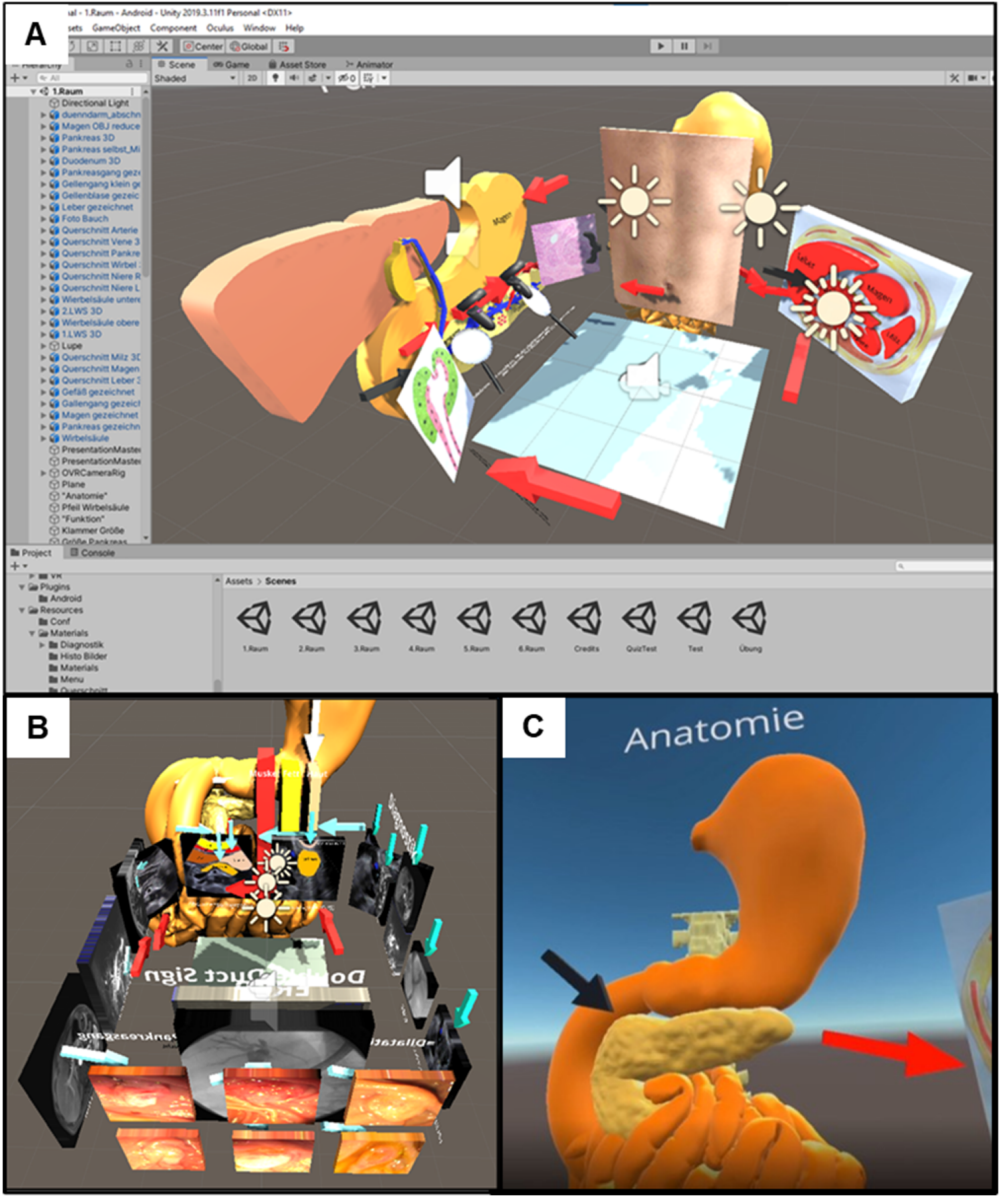
(A and B) Development environment of EVENT used for designing the virtual reality course. Images represent all elements in one of the VR rooms simultaneously displayed. C) Immersive view at the 3D contents generated inside the HMD.

EVENT is an open-source VR software, designed for preparing educational VR courses without programming skills. It can be embedded in the game engine Unity 3D (Unity Technologies, San Francisco, California, US), and it is available for download from the authors’ website.^16^ The software runs on common HMDs including HTC VIVE (HTC Corporation, Taoyuan, Taiwan), Meta Quest 1 and 2 (Meta Platforms, Inc., Menlo Park, California, US). By creating a VR environment with videos, audio files, 3D objects, tables, and images, users are allowed to immerse into a strong graphical interface and learn interactively at their own pace (Figure 1). Quizzes are integrated to reflect student learning progress (multiple choice, point quiz, sorting quiz). The lessons were created by a medical student with no programming experience. The installation of the application and the operation of EVENT are explained step by step using online videos (https://www.ukw.de/en/inexen/vr-applications/event-easy-vr-education-tool/). The lightweight HMD Meta Quest 1 was used as a headset, which has a horizontal field of view (FOV) of 104°, a vertical FOV of 100°, and an image resolution of 1440 × 1600 pixels. An investigator was always onsite to answer questions and provide technical assistance to students. The movement space for the personal VR environment was about 4 m² each.

### Study design

**Figure 2.**
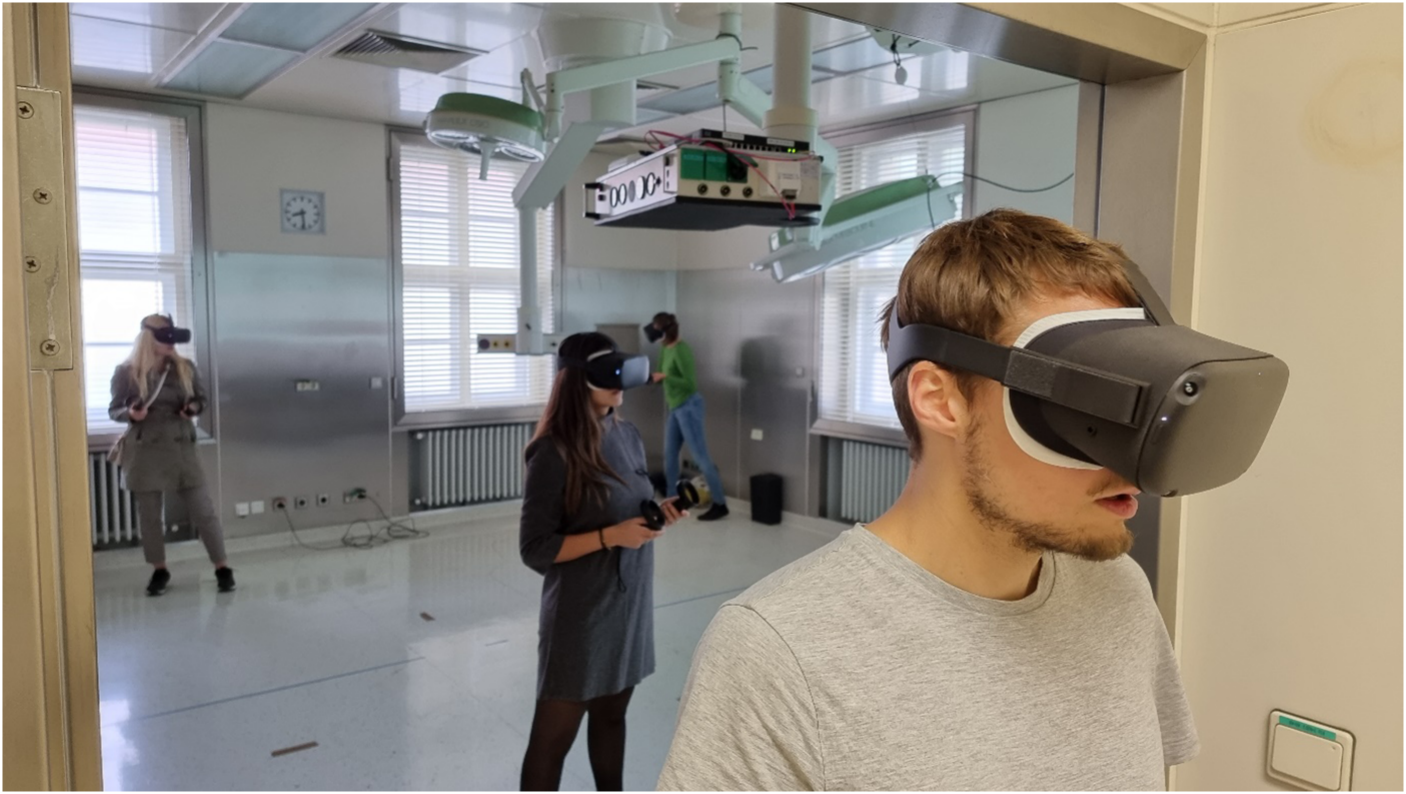
Participants during the virtual reality simulation course. Written consent for the publication of this photograph was obtained from all persons depicted.

The study was conducted as a single-arm prospective study from November 28, 2021 to December 12, 2022. All students from the sixth semester of the Clinical Practical Internship in Internal Medicine (KPIM 6) course at the University Hospital Würzburg, Germany, were included after signing informed consent. Voluntary students from the seventh and eighth semesters were also allowed to take part in the study after signing the informed consent form. Exclusion criteria for analysis were technical problems regarding the VR headset and incomplete questionnaire forms. EVENT was used for a 90-min medical student VR course on pancreatic cancer (Figure 2). Specific chapters of the course included “pancreatic anatomy and physiology,” “symptoms,” “diagnosis,” “risk factors and histopathology,” “treatment,” and “prognosis” of pancreatic cancer. Before and after the about 40 min lasting VR simulation, medical students completed a multiple-choice knowledge assessment with a maximum score of 10. The topics of the multiple-choice knowledge test were related to each chapter of the course. The chapters cover topics ranging from the anatomy and physiology of the pancreas to the prognosis of pancreatic cancer. The questions were developed by two board-certified gastroenterologists from the University Hospital Würzburg based on the learning objectives for the medical school second state examination in Germany. The participants also completed a questionnaire on VR experience and self-efficacy, which was answered on a five-point Likert scale (strongly agree to strongly disagree). The primary endpoint compared pre- and post-VR simulation test scores. Secondary endpoints that could affect learning growth and test results were included in the analysis. Usability was analyzed in the post-simulation questionnaire by the ten questions of the widely used system usability score (SUS).^21^ In addition, the students’ open answers regarding usability were analyzed. Seven questions of the post-simulation questionnaire addressed the subjective workload based on the National Aeronautics and Space Administration Task Load Index (NASA TLX).^22^

### Statistical analyses

Sum scores for test and self-efficacy questions were used to compare pre- and post-simulation results. The results of the multiple-choice tests were scored one point for no deviation and 0.5 points for up to two deviations from the correct answer. Normalized learning growth was calculated by the score of the post-simulation test minus the score of the pre-simulation test divided by the maximum possible learning growth. Threshold values were defined to transfer the questionnaire ratings into a dichotomous scale (favorable rating for the two most positive answer possibilities and unfavorable rating for the three least positive answer possibilities on the five-point Likert scale). Grouping of multiple questions aimed at one outcome in the questionnaires was achieved by dichotomization as described above, but for the average of all relevant questions. NASA TLX questions (10-point scale) were transferred accordingly, with favorable rating for the four most positive answer possibilities and unfavorable rating for the six least positive answer possibilities. Where appropriate, values are indicated with ± standard deviation. The distributions of quantitative and qualitative data are presented as the absolute and relative frequencies or averages (range), respectively. Fisher's two-sided exact test or Pearson's chi-squared test was performed on categorical variables, and Wilcoxon (paired) rank-sum test was performed on quantitative (paired) parameters. Spearman correlation was used for non-normally distributed numeric data. Statistical hypothesis testing was performed on the two-sided exploratory 0.05 significance levels. RStudio (version 4.0.2; R Foundation for Statistical Computing) was used for all statistical analyses.

## Results

### Baseline characteristics

One hundred and thirty-five medical students participated in the course. Eighteen participants were excluded due to a technical problem regarding acquisition of questionnaire data on the first-course day. No technical difficulties were encountered with the VR headset. Data of 117 medical senior-class students was included for analysis (Table 1). The participants of the VR experience completed the pre- and post-simulation test as well as the respective questionnaires as detailed in the Supplemental Material. 73.3% were female, and age ranged from 20 to 39 years of age with an average age of 23.5. 13.7% (16/117) had already completed professional training in the medical field and 31.6% (37/117) indicated having some prior knowledge on the medical topic taught in the course.

**Table 1.** Baseline characteristics of the participants.

**BASELINE CHARACTERISTICS**	**RELATIVE FREQUENCY IN % (ABSOLUTE FREQUENCY) OR AVERAGE (RANGE)**
Number of participants	117
Age in years	23.5 (20-39)
Sex, female	73.3% (85/116; 1 NA)
Third year of medical school	94.9% (111/117)
Completed professional training in the medical field	13.7% (16/117)
Prior knowledge on the medical topic	31.6% (37/117)
No prior experience with virtual reality	84.6% (99/117)
No prior experience with video games	83.8% (98/117)

Abbreviation: NA, not available.

### Primary endpoint

**Figure 3.**
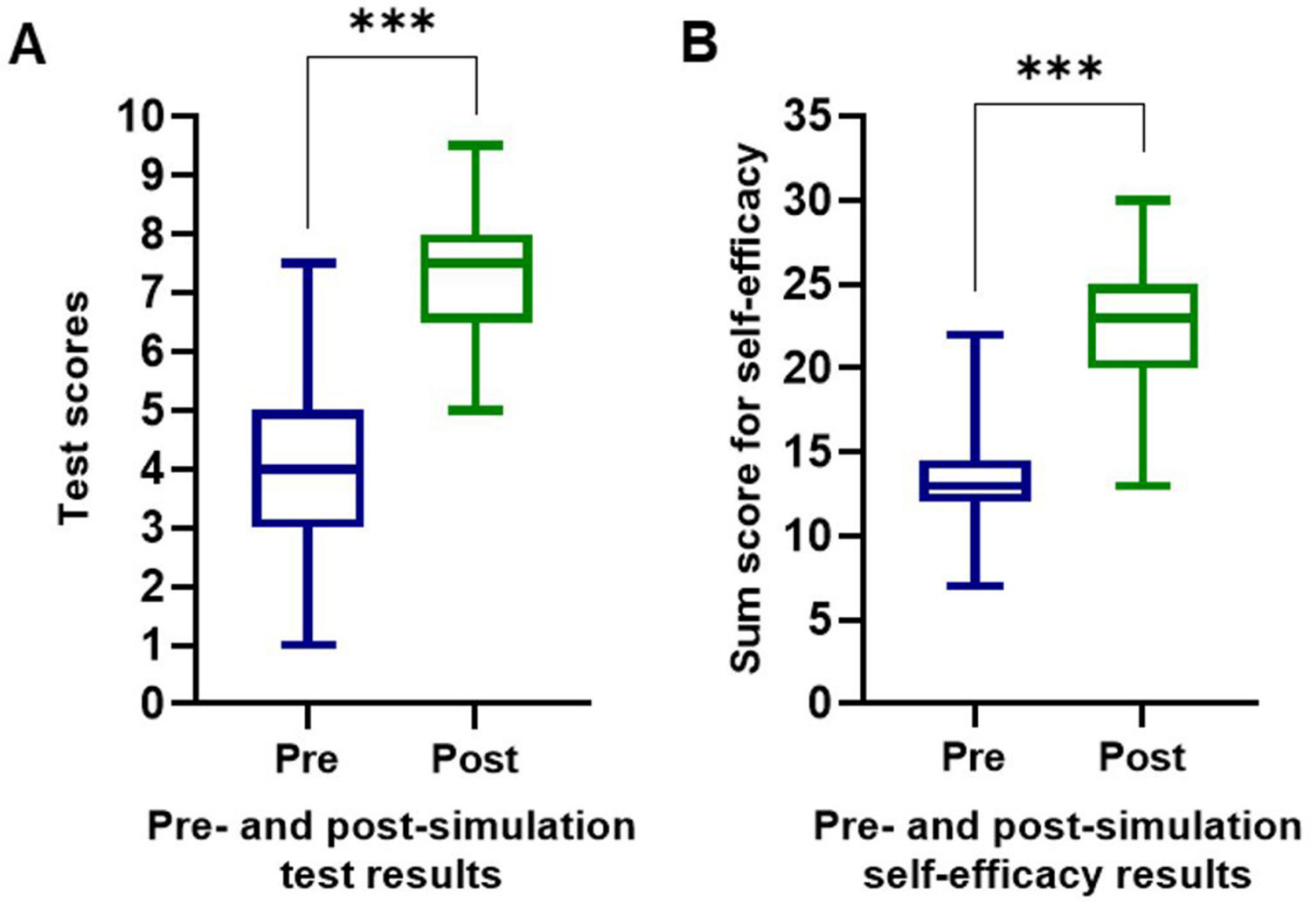
Pre- and post-simulation results of a multiple-choice knowledge assessment with a maximum score of 10, *P *< 0.001 (A) and self-efficacy evaluated by self-assessment of the knowledge about pancreatic cancer, *P *< 0.001 (B).

Pre-VR simulation test results on pancreatic cancer knowledge ranged from 1 (worst result) to 7.5 out of 10 (average 4.0 ± 1.2), whereas post-simulation test results ranged from 5 to 9.5 out of 10 (average 7.3 ± 1.1). Compared to pre-test scores, post-test scores improved by an average of 3.4 points (±1.6, 95% CI 3.1-3.7, *P *< 0.001 using the Wilcoxon paired rank-sum test, Figure 3A and Supplemental Figure S1A). The upper quartile included 15 students (12.8%) for the pre-simulation test and 27 students (23.1%) for the post-simulation test. The average normalized learning growth for all students was 55.9%.

### Secondary endpoints

84.6% (99/117) of students had no prior experience with VR, and 98/117 students reported to have never played video games (83.8%). 41.9% (49/117) of the students felt that they had good visual imagination, which was not influenced by gender or age (*P *= 1, *P *= 0.413). No minor or major technical problems occurred with VR simulation as assessed by the investigators. 19.7% (23/117) students indicated that they would need technical support to use the teaching unit.

76.9% (90/117) of the students were highly motivated to participate in the simulation. Regarding immersion, 88.0% (103/117) of the students indicated to be fully engaged by the simulation. During the simulation 35.9% [42/117] of all students reported no longer being aware of the real environment, which included people outside the simulation. It mattered to 50.4% (59/117) of students whether the simulation contained inconsistencies and/or contradictions, but only 9.4% (11/117) of the students noticed inconsistencies and/or contradictions during the actual simulations, which did not correlate to learning outcome. 65.8% (77/117) of students liked the VR design concept of the simulation. However, 18/117 (15.4%) struggled with nausea in the VR environment while moving in their ∼4 m² square field.

The above-described secondary endpoints (Table 1) of gender, age, prior knowledge regarding the medical subject, professional training completed in the medical field, poor spatial awareness, motivation, excitement from the immersive experience itself, or cyber-sickness had no major impact on test scores pre- or post VR simulation, or final ranking (top or bottom 25%) of test scores. Regarding prior VR experience, the 27 students whose post-VR simulation test scores ranked in the top 25% had no prior experience with VR.

Being stressed by the teaching unit itself without regard to the VR experience (42/117) significantly correlated to lower post-simulation results (average score for students feeling stressed by the teaching unit itself 7.0 points, average score for all others 7.5 points, *P *= 0.032) and ranking in the lower 75% (upper quartile: 4/27 [14.8%] stressed by the teaching unit itself, other quartiles 42.2% [38/90], *P *= 0.018).

Self-efficacy (a student's belief that he or she can successfully overcome challenging situations with his or her own resources) was analyzed by self-assessment of knowledge about pancreatic cancer topics, and increased significantly from pre- to post-simulation, see Supplemental Figure S1 and Supplemental Figure S1B (*P *< 0.001). Before the simulation, 70.1% (82/117) of the students considered VR to have a potential role in medical education. After VR simulation, 85.5% (100/117) answered these questions positively, which included 11 students who decreased their assessment of the potential role of VR.

The average system usability scale (SUS) score was 86.1 (45-100). 11 students scored <68 points and 3 students were excluded from SUS score analysis due to one or more missing answers for one of the ten questions. A radar chart (Supplemental Figure S2) depicts mean answer values to each SUS question. Students in the upper quartile of the post-simulation results all scored >68 on the SUS score, which indicated good usability based on a rating above 68 SUS points that significantly increased post-simulation test results (average for a SUS score <68 6.5 points, average for a SUS score >68 7.4 points, *P *= 0.016).

78.6% (92/117) of students reported finding the simulations to be enjoyable and not stressful, with no correlation to gender or age (*P *= 0.964, *P *= 0.449). 84.6% (99/117) of the students indicated that VR teaching increased interest in the medical topic, and the simulation motivated 70.9% (83/117) of the students to further pursue course contents. 85.5% (100/117) of the students reported that they felt they did well in this simulation with a tendency for younger students to evaluate their performance more likely as satisfying (average 23.2 years) compared to older students (average 24.9 years, *P *= 0.072). 4 students indicated that they felt pressed for time to complete the task (3.4%). 2 students found the teaching unit to be frustrating (1.7%). 115/117 (98.3%) reported that they successfully completed the teaching unit on self-assessment.

In the qualitative evaluation, participants suggested some practical ways to improve VR teaching. These included equalizing the audio volume of all videos, using more 3D effects for the images, being able to control the speed of the videos, using headphones when in a room with other students, and having enough space in the room. 81.2% (95/117) students wished for more VR teaching opportunities in medical school.

## Discussion

VR has matured in terms of content and methodology over the past decades and can now be generated without extensive effort or prior knowledge, which is evident by the open-source software EVENT. While a VR environment by itself does not automatically lead to an improved learning outcome, an appropriate scenario design paired with a well-programmed software appears to provide increased learning growth in this study.

However, factors such as student demographics, technical challenges, little prior experience with VR or video games, poor spatial awareness, low motivation, excitement from the immersive experience itself, or cyber-sickness may negatively affect learning outcomes. We specifically targeted these possible influences with the post-test simulation questionnaire.

Age (20-39) or gender did not influence the test outcomes or student-perceived outcomes in our study. There were no technical challenges before or during the simulation. However, technical support should always be available to students, which was confirmed by 19.6% of the participants in the post-simulation questionnaire reporting a desire for technical support. One student reported too many inconsistencies during the teaching course, and 9.4% noticed inconsistencies and/or contradictions in the simulation, which did not influence their learning experience. However, half of the participants noted that inconsistencies in a VR simulation would bother them in principle, which underlines the importance of an easy-to-use software for preparing a teaching course.

About four-fifths of the students had no prior experience with VR. We suspected that this might adversely affect how they progressed in learning. Interestingly, this does not seem to have an impact on the VR-based learning outcome in our study, since all students in the upper 25% of the post-simulation test indicated no prior experience with VR. This finding could be promising to reach and to engage many students with this teaching modality.

More than three-quarters of the students were highly motivated to participate in the simulation, but low motivation did not correlate with lower learning gains or poorer test scores after the simulation.

Despite a low percentage of students feeling skilled in imagination (41.9%), this feeling did not correlate to better test outcomes, learning growth, or enjoying the simulation. 88.0% of students reported being fully engaged by the simulation, but this appeared to be a balanced level of excitement that did not negatively impact learning outcomes as previously reported by Jensen et al.^2^ However, feeling stressed by the course itself, without regard to the VR-experience, correlated significantly with lower post-simulation test scores (0.5 points lower, on average), which possibly indicates that performance pressure needs to be addressed.

Cyber-sickness can be a serious barrier to learning success in a VR environment, which may force study participants to pause,^23^ or even dropout of the study.^24^ In our study, 18/117 students (15.4%) reported cyber-sickness, which did not significantly correlate with learning outcomes. In recent years, cyber-sickness seems to be mitigated by improved graphical representation in the VR environment, and this trend is expected to increase in the future.^25,26^

Most students indicated a balanced level of immersion that allowed the interaction with the VR environment while still being aware of the actual surroundings, which include people outside the simulation. Several important factors impairing immersion have been described, such as jerky animation,^27^ sitting in comparison to standing up,^24^ or distracting the user by 3D sounds or displaying virtual hands as the user's hands.^28^ These were considered for the preparation of this study simulation.

We found in this study that the mental/physical workload was balanced and that the percentage of students who feel under time pressure was low (3.4%). However, we see further potential to reduce the perception of stress by the teaching unit itself (35.9%) by limiting performance pressure.

The SUS score, on average 86.1 (45-100), corresponded to an excellent outcome for user-friendliness^29–31.^ Easier adjustment to the device, and the simulation, significantly correlated to better learning outcomes, which stresses the importance of user-friendliness. Overall, 78.6% of students reported that they found the simulations enjoyable and stress-free, and the percentage of students interested in more VR simulations in medical school increased by 20%. The main study limitation is the lack of a control group, preventing the evaluation of superiority, equality, or inferiority of the VR simulations compared to the regular teaching format. Furthermore, no sample size calculation was carried out before the start of the study, as all students in the sixth semester were to be included. In addition, demographic factors other than gender and age have not been assessed and might have influenced the study outcome. With the exception of NASA-TLX and SUS, the questionnaires we used have not been validated. The pre-test and post-test had to include the same questions, but students were not allowed to use any other tools besides the VR unit to look up the answers.

## Conclusion

In conclusion, we present a freely available software that allows for the development of VR teaching lessons without programming skills. Usage of one of those lessons presented a significant knowledge gain. Additionally, the application was highly valued by students, even for those without VR experience. Future comparative studies should include e-learning on a 2D monitor with the same course content as a control group to evaluate the effect of full immersion. Another important comparison would be with an interactive seminar, which is easier to implement due to lower hardware requirements, but requires more staff. As part of these comparative studies, the long-term retention of acquired knowledge should be analyzed.

## Supplemental Material

sj-docx-1-mde-10.1177_23821205241242220 - Supplemental material for Evaluation of a Virtual Reality-Based Open Educational Resource Software Supplemental material, sj-docx-1-mde-10.1177_23821205241242220 for Evaluation of a Virtual Reality-Based Open Educational Resource Software by Silvia Würstle, Lisa-Marie Spanke, Niklas Mehlhase, Gail Stanley, Jonathan Koff, Stavros Dimitriadis, Sarah König and Alexander Hann in Journal of Medical Education and Curricular Development

sj-pdf-2-mde-10.1177_23821205241242220 - Supplemental material for Evaluation of a Virtual Reality-Based Open Educational Resource Software Supplemental material, sj-pdf-2-mde-10.1177_23821205241242220 for Evaluation of a Virtual Reality-Based Open Educational Resource Software by Silvia Würstle, Lisa-Marie Spanke, Niklas Mehlhase, Gail Stanley, Jonathan Koff, Stavros Dimitriadis, Sarah König and Alexander Hann in Journal of Medical Education and Curricular Development

sj-xlsx-3-mde-10.1177_23821205241242220 - Supplemental material for Evaluation of a Virtual Reality-Based Open Educational Resource Software Supplemental material, sj-xlsx-3-mde-10.1177_23821205241242220 for Evaluation of a Virtual Reality-Based Open Educational Resource Software by Silvia Würstle, Lisa-Marie Spanke, Niklas Mehlhase, Gail Stanley, Jonathan Koff, Stavros Dimitriadis, Sarah König and Alexander Hann in Journal of Medical Education and Curricular Development
