# Supplementary material for: Evaluation of a Virtual Reality-Based Open Educational Resource Software
Source: J Med Educ Curric Dev. 2024 Apr 2;11:23821205241242220. doi: 10.1177/23821205241242220 (PMC10989036; doi:10.1177/23821205241242220)

**Manuscript: Evaluation of a virtual reality based open educational resource software**

**Supplementary Material:**

- Questionnaire before and after the VR experience (PDF files).
- Questionnaire answers before and after the VR experience (Excel-file).
- Supplementary Figure 1 and 2, see below.

**Supplementary Figures:**

**Supplementary Figure 1:** Pre- and post-simulation test and self-efficacy results depicting all datapoints of Figure 1 of the manuscript. **A** Test results. **B** Self-efficacy results.

**
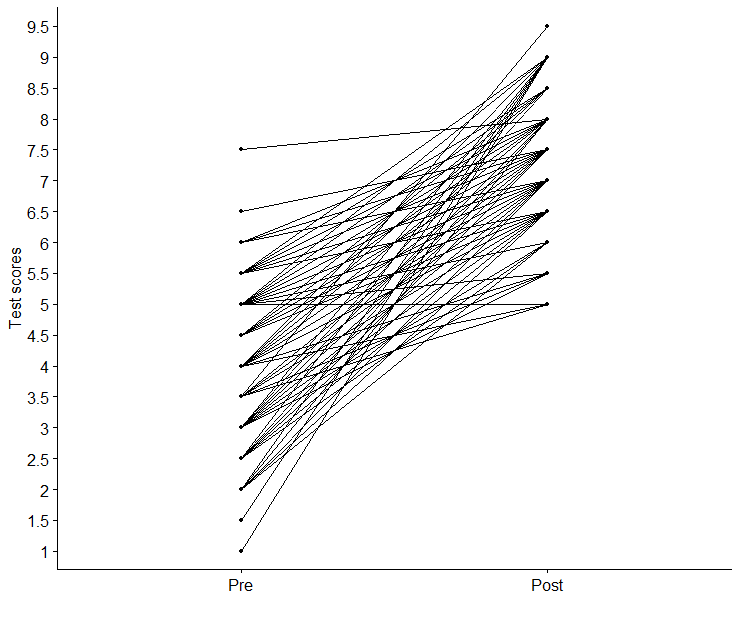
A**

**
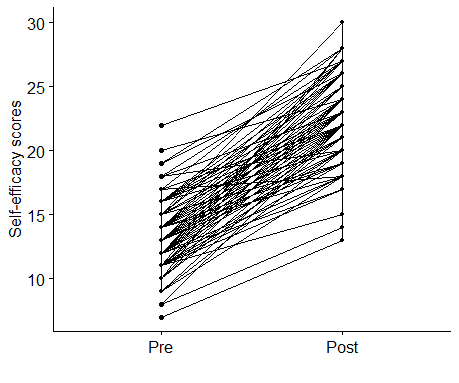
B**

**Supplementary Figure 2**: Radar chart depicting mean answer values to all SUS related questions. SUS related questions are detailed in the Supplementary Material, Questionnaire after the VR experience (PDF file), Questions 3.1-3.10. The answers are rated on a scale of 1 to 5, with 1 being full agreement and 5 being full disagreement.


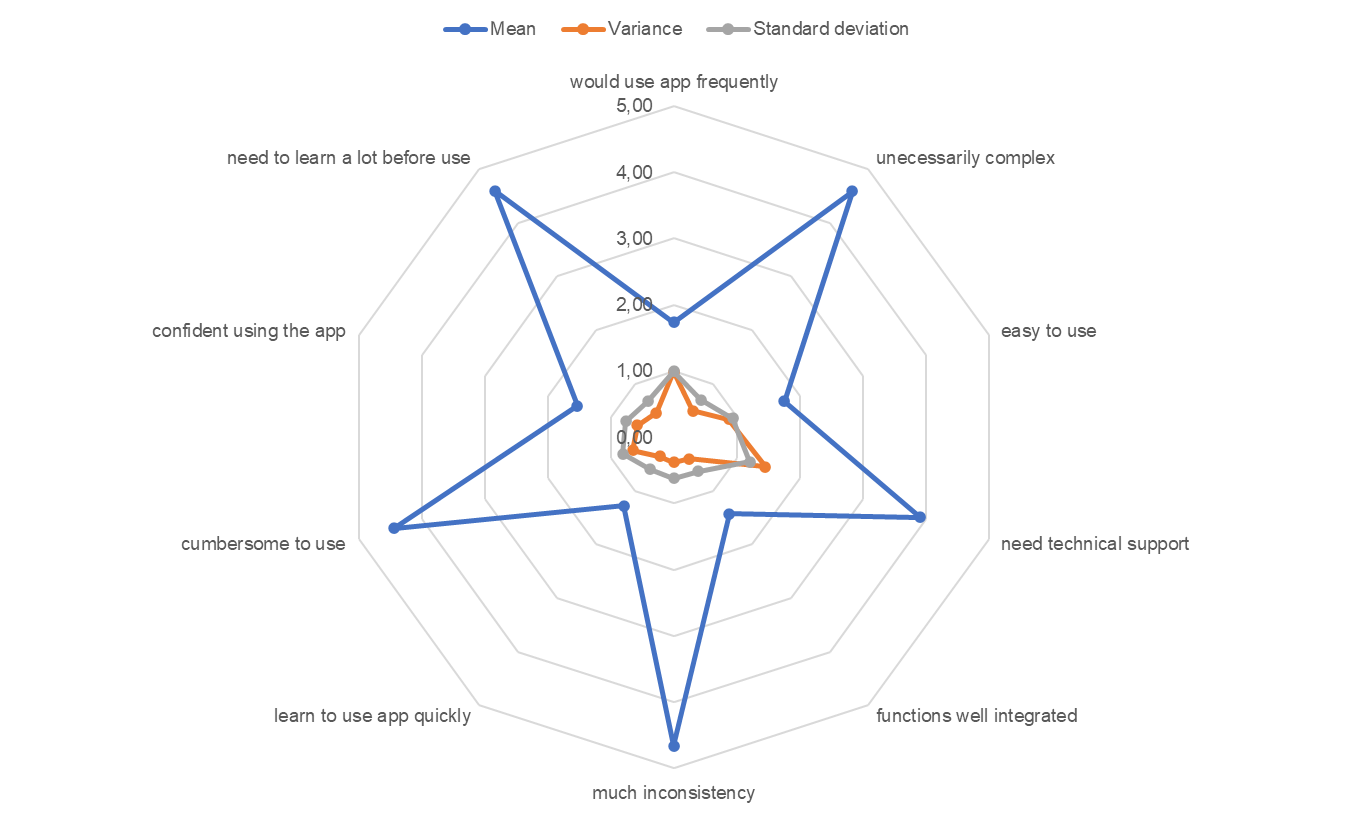

Supplement: sj-docx-1-mde-10.1177_23821205241242220 - Supplemental material for Evaluation of a Virtual Reality-Based Open Educational Resource Software [file sj-docx-1-mde-10.1177_23821205241242220.docx]
