# Supplementary material for: Evaluation of a Virtual Reality-Based Open Educational Resource Software
Source: J Med Educ Curric Dev. 2024 Apr 2;11:23821205241242220. doi: 10.1177/23821205241242220 (PMC10989036; doi:10.1177/23821205241242220)
Supplement: sj-pdf-2-mde-10.1177_23821205241242220 - Supplemental material for Evaluation of a Virtual Reality-Based Open Educational Resource Software [file sj-pdf-2-mde-10.1177_23821205241242220.pdf]

Medizinische Fakultät — Lisa Spanke

Qualitätssicherung der Lehre in der Medizin — please complete the questionnaire before the VR simulation

Bitte so markieren: ☐ ☒ ☐ ☐ ☐

Korrektur: ☐ ■ ☐ ☒ ☐

1. Basics

1.1 Participant number:

1.2 Semester of Study: ☐ 1. ☐ 2. ☐ 3. ☐ 4. ☐ 5. ☐ 6. ☐ 7. ☐ 8. ☐ 9. ☐ > 10.

1.3 Age:

1.4 Gender: ☐ f ☐ m ☐ diverse

1.5 Do you already have a completed professional training in the medical field? ☐ yes ☐ no

1.6 How often do you play first-person action games (e.g. Minecraft, Counter Strike or Video story games like Subnautica)? never ☐ ☐ ☐ ☐ ☐ frequent

2. Self-efficacy

2.1	I am well versed in the anatomy of the pancreas.	agree ☐	☐	☐	☐	☐ dissagree
2.2	I have a good understanding of the physiology of the pancreas.	agree ☐	☐	☐	☐	☐ dissagree
2.3	I know different imaging modalities to evaluate the pancreas.	agree ☐	☐	☐	☐	☐ dissagree
2.4	I feel confident in recommending further diagnostics to a patient with suspected pancreatic cancer.	agree ☐	☐	☐	☐	☐ dissagree
2.5	I feel confident in recommending therapy to a patient diagnosed with pancreatic cancer.	agree ☐	☐	☐	☐	☐ dissagree
2.6	I feel well prepared for an exam based on my current knowledge of pancreatic cancer.	agree ☐	☐	☐	☐	☐ dissagree

3. VR Pre-Experience

3.1 I have gained experience with VR over the past year: ☐ 1x ☐ 2x ☐ > 3x ☐ disagree

4. VR General

4.1 Have you ever had a VR teaching session ? ☐ yes ☐ no

4.2	I see great potential for the use of VR in student teaching.	agree ☐	☐	☐	☐	☐ dissagree
4.3	I would like to see more VR offerings in medical school.	agree ☐	☐	☐	☐	☐ dissagree

5. MC-Questions

5. MC-Questions [Fortsetzung]

5.1 The mesenteric vascular axis ...
(Several answers are correct)

- ☐ consists of superior mesenteric artery and vein
- ☐ consists of common mesenteric artery and vein
- ☐ arises from the abdominal aorta at the level of the thoracic vertebral body 12
- ☐ is enclosed by the pancreatic uncinate process
- ☐ is accompanied by branches of the celiac plexus

5.2 The excretory ducts of the pancreas ...
(Several answers are correct)

- ☐ are called duct of Wirsung and duct of Santorini
- ☐ are called duct of Wirsung and ductus Botalli
- ☐ and the common bile duct, drain into the large papilla of the duodenum.
- ☐ are the origin of the majority of pancreatic cancers

5.3 The most common pathologic morphology of pancreatic cancer is the ...
(1 answer is correct)

- ☐ Acinar cell carcinoma
- ☐ Adenocarcinoma
- ☐ Squamous cell carcinoma
- ☐ Signet ring carcinoma
- ☐ Carcinoma of giant cell type

5.4 **Early clinical signs** of pancreatic cancer include...
(1 answer is correct)

- ☐ epigastric pain due to perineural infiltration of tumor cells
- ☐ Murphy's sign
- ☐ weight loss
- ☐ exocrine pancreatic insufficiency
- ☐ pancreoprivic diabetes

5.5 One of the clinical late signs of pancreatic cancer is the Courvoisier sign. This is clinically characterized ...
(1 answer is correct)

- ☐ by a palpable bulging **painless** gallbladder
- ☐ by a palpable bulging **painful** gallbladder
- ☐ by a palpable tumor in the upper abdomen at the level of the lumbar vertebral body 2
- ☐ by the triad of jaundice, cachexia and diabetes mellitus
- ☐ by chologenic diarrhea with discolored stool

5. MC-Questions [Fortsetzung]

5.6 Numerous diagnostic procedures are available to diagnose pancreatic cancer:
(1 answer is correct)

- ☐ On contrast-enhanced CT, pancreatic carcinoma presents as hypodense in the early arterial phase
- ☐ The majority of of pancreatic carcinomas show up in the tail of the pancreas according to the organ volume fraction
- ☐ Endosonography reduces the distance between the transducer and the target organ is reduced, so that a higher spatial resolution is achieved by a long-wave sound spectrum
- ☐ MRI allows an additional assessment of the pancreatic duct structures by evaluation of the MRCP sequence. A double-duct sign is a simultaneous dilatation of the duct of Wirsung and the duct of Santorini
- ☐ ERCP is considered a standard diagnostic procedure in the initial diagnosis of pancreatic cancer

5.7 What are risk factors for developing pancreatic cancer:
(Several answers are correct)

- ☐ diabetes mellitus
- ☐ nicotine consumption
- ☐ alcohol consumption
- ☐ hereditary pancreatitis e.g. as a result of PRSS1 mutation
- ☐ MEN 1

5.8 Which statements about the prognosis of pancreatic carcinoma are correct:
(Several answers are correct)

- ☐ Pancreatic cancer has a poor prognosis, with a median 5 year survival rate <10%
- ☐ Thanks to diagnostic and therapeutic medical progress, the 5 year survival rate could be significantly increased in the last 15 years
- ☐ At initial diagnosis, 80% of pancreatic cancers are already locally advanced or metastatic and thus outside of curative treatment options
- ☐ Complete curative resection opens up a long-term prognosis with a 5 year survival rate of >50%.

5.9 The standard surgical procedure for resection of pancreatic head carcinoma is pylorus-preserving partial pancreaticoduodenectomy. Removed are ...
(Several answers are correct)

- ☐ pancreatic head
- ☐ duodenum
- ☐ distal stomach
- ☐ gall bladder
- ☐ parts of the left liver lobe

5. MC-Questions [Fortsetzung]

5.10 Adjuvant (postoperative) chemotherapy ...
(Several answers are correct)

- ☐ should be administered to all patients
- ☐ is only required for patients with lymph node metastases
- ☐ Therapy standard is Folfox (5-flourouracil+ oxaliplatin) for 6 months
- ☐ One treatment option is Gemcitabine over 6 months
- ☐ One treatment option is FOLFIRINOX (5-FU+ irinitecan+ oxaliplatin) for 6 months.

Medizinische Fakultät | Lisa Spanke

Qualitätssicherung der Lehre in der Medizin | please fill out the questionnaire after the VR simulation

Bitte so markieren: ☐ ☒ ☐ ☐ ☐

Korrektur: ☐ ■ ☐ ☒ ☐

1. Basics

1.1 Matriculation number:

1.2 Participant number:

2. Self-efficacy

2.1	I am well versed in the anatomy of the pancreas.	agree ☐	☐	☐	☐	☐	dissagree
2.2	I have a good understanding of the physiology of the pancreas.	agree ☐	☐	☐	☐	☐	dissagree
2.3	I know different imaging modalities to evaluate the pancreas.	agree ☐	☐	☐	☐	☐	dissagree
2.4	I feel confident in recommending further diagnostics to a patient with suspected pancreatic cancer.	agree ☐	☐	☐	☐	☐	dissagree
2.5	I feel confident in making a treatment recommendation for a diagnosed pancreatic cancer.	agree ☐	☐	☐	☐	☐	dissagree
2.6	I feel well prepared for an exam by my current knowledge regarding pancreatic cancer.	agree ☐	☐	☐	☐	☐	dissagree

3. Usability - SUS Score

3.1	I can very well imagine using the teaching unit on a regular basis.	agree ☐	☐	☐	☐	☐	dissagree
3.2	I feel the teaching unit is unnecessarily complex.	agree ☐	☐	☐	☐	☐	dissagree
3.3	I find the teaching unit easy to use.	agree ☐	☐	☐	☐	☐	dissagree
3.4	I think I would need technical support to use the teaching unit.	agree ☐	☐	☐	☐	☐	dissagree
3.5	I find that the various functions are well integrated into the teaching unit.	agree ☐	☐	☐	☐	☐	dissagree
3.6	I think there are too many inconsistencies in the teaching unit.	agree ☐	☐	☐	☐	☐	dissagree
3.7	I imagine most people learn to master the teaching unit quickly.	agree ☐	☐	☐	☐	☐	dissagree
3.8	I find the operation very cumbersome.	agree ☐	☐	☐	☐	☐	dissagree
3.9	I felt safe using the teaching unit.	agree ☐	☐	☐	☐	☐	dissagree
3.10	I had to learn a lot of things before I could work with the teaching unit.	agree ☐	☐	☐	☐	☐	dissagree

4. VR General

4.1	I see great potential for the use of VR in student teaching.	agree ☐	☐	☐	☐	☐	dissagree
4.2	I would like to see more VR offerings in medical school.	agree ☐	☐	☐	☐	☐	dissagree

5. NASA TLX Score

5.1 The teaching unit challenged me mentally. agree ☐ ☐ ☐ ☐ ☐ ☐ ☐ ☐ ☐ ☐ dissagree

5. NASA TLX Score [Fortsetzung]

5.2	The teaching unit challenged me physically.	agree	☐	☐	☐	☐	☐	☐	☐	☐	☐	☐	dissagree
5.3	I was under time pressure to complete the tasks.	agree	☐	☐	☐	☐	☐	☐	☐	☐	☐	☐	dissagree
5.4	I struggled with nausea in the VR environment.	agree	☐	☐	☐	☐	☐	☐	☐	☐	☐	☐	dissagree
5.5	I have successfully mastered the teaching unit.	agree	☐	☐	☐	☐	☐	☐	☐	☐	☐	☐	dissagree
5.6	I was stressed by the teaching unit.	agree	☐	☐	☐	☐	☐	☐	☐	☐	☐	☐	dissagree
5.7	The teaching session was frustrating.	agree	☐	☐	☐	☐	☐	☐	☐	☐	☐	☐	dissagree

6. Interest

6.1	The teaching unit raised my interest in the subject area.	agree	☐	☐	☐	☐	☐	dissagree
6.2	In this teaching unit, I learned things that excite me.	agree	☐	☐	☐	☐	☐	dissagree
6.3	The teaching unit has been very responsive to my experience.	agree	☐	☐	☐	☐	☐	dissagree

7. Attention

7.1	The simulation completely absorbed my attention.	agree	☐	☐	☐	☐	☐	dissagree
7.2	My perception has turned to the simulation as if by itself.	agree	☐	☐	☐	☐	☐	dissagree
7.3	I didn't even notice how time was passing.	agree	☐	☐	☐	☐	☐	dissagree

8. Involvement

8.1	I was no longer aware of my real environment during the simulation.	agree	☐	☐	☐	☐	☐	dissagree
8.2	I stopped paying attention to other people present (e. g.fellow students, tutors, lecturers) who were not part of the simulation.	agree	☐	☐	☐	☐	☐	dissagree

9. Spatial perception

9.1	I could well imagine the environment depicted or meant.	agree	☐	☐	☐	☐	☐	dissagree
9.2	I had a clear idea of what was happening.	agree	☐	☐	☐	☐	☐	dissagree
9.3	I still have a vivid picture of the situation in my mind.	agree	☐	☐	☐	☐	☐	dissagree

10. Self-location

10.1	I felt as if I myself had been present in the simulation on site.	agree	☐	☐	☐	☐	☐	dissagree

11. Possibilities for action

11.1	I had the feeling of being able to act actively in the simulation.	agree	☐	☐	☐	☐	☐	dissagree
11.2	I had the impression of being able to immerse myself in the action.	agree	☐	☐	☐	☐	☐	dissagree

12. Suspension of disbelief / willingness to engage

12.1	I noticed inconsistencies and/or contradictions in the simulation.	agree	☐	☐	☐	☐	☐	dissagree
12.2	It did not matter to me whether the simulation contained errors and/or contradictions.	agree	☐	☐	☐	☐	☐	dissagree

13. Open questions

13. Open questions [Fortsetzung]

13.1 Do you have any suggestions for improving the simulation?

13.2 If there was a moment when you were unintentionally brought back from the simulation, what was the trigger for that?

13.3 What exactly completely captivated you in the simulation?

13.4 What else would you like to share with us ...

Assessments of the learning process and learning success :

14. Content complexity and challenge

14.1 I felt I was up to the challenge in the simulation.	agree ☐	☐	☐	☐	☐ dissagree
14.2 I was tense during the simulation.	agree ☐	☐	☐	☐	☐ dissagree
14.3 I enjoyed the simulation.	agree ☐	☐	☐	☐	☐ dissagree
14.4 I was stressed because I felt I had no control over the situation.	agree ☐	☐	☐	☐	☐ dissagree
14.5 I had enough prior knowledge to work on the simulation.	agree ☐	☐	☐	☐	☐ dissagree

15. Subjective learning success

15.1 The simulation helps me to cope better in real situations involving this issue.	agree ☐	☐	☐	☐	☐ dissagree
15.2 The simulation is a good tool for the acquisition of medical skills.	agree ☐	☐	☐	☐	☐ dissagree

16. Perceived competence

16.1 I think I did well in this simulation.	agree ☐	☐	☐	☐	☐ dissagree
16.2 I am satisfied with my performance.	agree ☐	☐	☐	☐	☐ dissagree

Motivation and interest :

17. Motivation

17.1 I was looking forward to the course.	agree ☐	☐	☐	☐	☐ dissagree
17.2 What I heard in advance about the course motivated me.	agree ☐	☐	☐	☐	☐ dissagree

17. Motivation [Fortsetzung]

17.3 I was ready to engage in the simulation. agree ☐ ☐ ☐ ☐ ☐ dissagree

18. Area specific interest

18.1 I am interested in the subject of the course. agree ☐ ☐ ☐ ☐ ☐ dissagree

18.2 I already had prior knowledge on this subject. agree ☐ ☐ ☐ ☐ ☐ dissagree

18.3 The simulation motivates me to further deepen the contents of the course. agree ☐ ☐ ☐ ☐ ☐ dissagree

Visual imagination :

19. Visual imagination

19.1 When reading, I often have a detailed picture of the described environment in my mind's eye. agree ☐ ☐ ☐ ☐ ☐ dissagree

19.2 When someone describes a path to me, I see it "like a movie" in front of me. agree ☐ ☐ ☐ ☐ ☐ dissagree

19.3 When someone describes a room to me, it is very easy for me to imagine it clearly. agree ☐ ☐ ☐ ☐ ☐ dissagree

19.4 If a picture shows only a section of a room, I can clearly imagine the rest of the room. agree ☐ ☐ ☐ ☐ ☐ dissagree

20. MC-Questions

20.1 The mesenteric vascular axis ...
(Several answers are correct)

- ☐ consists of superior mesenteric artery and vein
- ☐ consists of common mesenteric artery and vein
- ☐ arises from the abdominal aorta at the level of the thoracic vertebral body 12
- ☐ is enclosed by the pancreatic uncinate process
- ☐ is accompanied by branches of the celiac plexus

20.2 The excretory ducts of the pancreas ...
(Several answers are correct)

- ☐ are called duct of Wirsung and duct of Santorini
- ☐ are called duct of Wirsung and ductus Botalli
- ☐ flow together with the common bile duct into the major duodenal papilla
- ☐ are the origin of the majority of pancreatic cancers

20.3 The most common pathologic morphology of pancreatic cancer is the ...
(1 answer is correct)

- ☐ Acinar cell carcinoma
- ☐ Adenocarcinoma
- ☐ Squamous cell carcinoma
- ☐ Signet ring carcinoma
- ☐ Carcinoma of giant cell type

20.4 **Early clinical signs** of pancreatic cancer include...
(1 answer is correct)

- ☐ epigastric pain due to perineural infiltration of tumor cells
- ☐ Murphy's sign
- ☐ weight loss
- ☐ exocrine pancreatic insufficiency
- ☐ pancreoprivic diabetes

20. MC-Questions [Fortsetzung]

20.5 One of the clinical late signs of pancreatic cancer is the Courvoisier sign. This is clinically characterized ...
(1 answer is correct)

- ☐ by a palpable bulging **painless** gallbladder
- ☐ by a palpable bulging **painful** gallbladder
- ☐ by a palpable tumor in the upper abdomen at the level of the lumbar vertebral body 2
- ☐ by the triad of jaundice, cachexia and diabetes mellitus
- ☐ by chologenic diarrhea with discolored stool

20.6 Numerous diagnostic procedures are available to diagnose pancreatic cancer:
(1 answer is correct)

- ☐ On contrast-enhanced CT, pancreatic carcinoma presents as hypodense in the early arterial phase
- ☐ The majority of of pancreatic carcinomas show up in the tail of the pancreas according to the organ volume fraction
- ☐ Endosonography reduces the distance between the transducer and the target organ target organ is reduced, so that a higher spatial resolution is achieved by a long-wave sound spectrum
- ☐ MRI allows an additional assessment of the pancreatic duct structures by evaluation of the MRCP sequence. A double-duct sign is a simultaneous dilatation of the duct of Wirsung and the duct of Santorini
- ☐ ERCP is considered a standard diagnostic procedure in the initial diagnosis of pancreatic cancer

20.7 What are risk factors for developing pancreatic cancer:
(Several answers are correct)

- ☐ diabetes mellitus
- ☐ nicotine consumption
- ☐ alcohol consumption
- ☐ hereditary pancreatitis e.g. as a result of PRSS1 mutation
- ☐ MEN 1

20.8 Which statements about the prognosis of pancreatic carcinoma are correct:
(Several answers are correct)

- ☐ Pancreatic cancer has a poor prognosis, with a median 5 year survival rate <10%
- ☐ Thanks to diagnostic and therapeutic medical progress, the 5 year survival rate could be significantly increased in the last 15 years
- ☐ At initial diagnosis, 80% of pancreatic cancers are already locally advanced or metastatic and thus outside of curative treatment options
- ☐ Complete curative resection opens up a long-term prognosis with a 5 year survival rate of >50%.

20. MC-Questions [Fortsetzung]

20.9 The standard surgical procedure for resection of pancreatic head carcinoma is pylorus-preserving partial pancreaticoduodenectomy. Removed are ...
(Several answers are correct)

- ☐ pancreatic head
- ☐ duodenum
- ☐ distal stomach
- ☐ gall bladder
- ☐ parts of the left liver lobe

20.10 Adjuvant (postoperative) chemotherapy ...
(Several answers are correct)

- ☐ should be administered to all patients
- ☐ is only required for patients with lymph node metastases
- ☐ Therapy standard is Folfox (5-flourouracil+ oxaliplatin) for 6 months
- ☐ One treatment option is Gemcitabine over 6 months
- ☐ One treatment option is FOLFIRINOX (5-FU+ irinitecan+ oxaliplatin) for 6 months.
